# Effect of Initial Conformation on the Starch Biopolymer Film Formation Studied by NMR

**DOI:** 10.3390/molecules25051227

**Published:** 2020-03-09

**Authors:** Sushanta Ghoshal, Carlos Mattea, Paul Denner, Siegfried Stapf

**Affiliations:** Department of Technical Physics II/Polymer Physics, Institute of Physics, Faculty of Mathematics and Natural Science, Ilmenau University of Technology, PO Box 10 05 65, D-98684 Ilmenau, Germany; paul.denner@tu-ilmenau.de (P.D.); siegfried.stapf@tu-ilmenau.de (S.S.)

**Keywords:** starch biopolymer, porous biopolymers, film formation, NMR, dynamic heterogeneity

## Abstract

The formation of a rigid porous biopolymer scaffold from aqueous samples of 1% *w/v* (suspension) and 5% *w/v* (gel) corn starch was studied using optical and nuclear magnetic resonance (NMR) techniques. The drying process of these systems was observed using a single-sided NMR scanner by application of the Carr–Purcell–Meiboom–Gill pulse sequence at different layer positions. The echo decays were analyzed and spin–spin relaxation times (*T_2_*) were obtained for each layer. From the depth dependent *T_2_* relaxation time study, it was found that the molecular mobility of water within the forming porous matrix of these two samples varied notably at different stages of film formation. At an intermediate stage, a gradual decrease in mobility of the emulsion sample towards the air–sample interface was observed, while the gel sample remained homogeneous all along the sample height. At a later stage of drying, heterogeneity in the molecular dynamics was observed in both samples showing low mobility at the bottom part of the sample. A wide-angle X-ray diffraction study confirmed that the structural heterogeneity persisted in the final film obtained from the 5% corn starch aqueous sample, whereas the film obtained from the 1% corn starch in water was structurally homogeneous.

## 1. Introduction

Starch is the second most abundant natural biopolymer after cellulose from plant origin. The glucose (D-glucopyranose) unit is the only monomer present in starch with two main constituents, namely, linear amylose and highly branched amylopectin. There are two connecting glycosidic α−(1→4)  and α-(1→6) linkages that construct the starch polymer backbone and branching points, respectively. Starch can be found in various parts of a plant such as seeds, leaves, roots, tubers, and the fruit pulps. The application of starch films is steadily increasing including biodegradable packaging materials, as an alternative coating material in the food industry, barrier against gases (O_2_, CO_2_), as a carrier in the pharmaceutical industry, etc. [1,2].

The ratio of amylose to amylopectin in the starch granules varies with the plant source. However, the amylose content in the commercially available starch granules is 20%–30%. Native starch granules have a crystallinity which varies from 12%–45%. Amylopectin mainly forms the semi-crystalline zone in the starch granules which consists of an ordered double helical lamellar structure and rigid amorphous branching zones. Some of the amylose may take part in this semi-crystalline zone by the formation of double helices with amylopectin side chains. On the other hand, both amylose and amylopectin contribute to the randomly ordered amorphous zone. Depending on the origin, native starch has three crystalline patterns. For example, most cereal starches have the so-called A-type pattern, whereas the B-type pattern appears in some tuber and cereal starches rich in amylose. Legume starches generally give a C-type pattern. The A-type pattern has relatively more dense packed double helices than the B-type [3,4,5].

The starch granule is composed of three different regions which are the amorphous region, crystalline lamellae, and the amorphous growth ring. At room temperature, a starch granule is not soluble in water because of the stable semi-crystalline structure. However, it undergoes limited swelling (reversible), presumably due to the hydration and swelling of the amorphous regions. It is known that amylopectin, due to the fact of its highly branched open structure, allows access to hydrogen-bonding solvent molecules. Consequently, it is more disrupted in water or another solvent able to form hydrogen bonding than amylose, which has a tightly packed structure. The interaction of starch granules with hot water is different to water at room temperature. For example, when the starch granules are heated in excess water beyond a critical temperature depending on the type of starch, an irreversible change is taking place known as gelatinization [3]. This involves swelling of the granules, crystallite melting, and solubilization. During swelling, the amorphous phase of granular starch is fully plasticized by the solvent and amylose begins to leach. This process is influenced by the increase in temperature. Depending on the initial concentration of starch in water, a precipitation or a white elastic gel (prepared from a concentrated system) can be obtained when the system is left to rest and cools down to room temperature [6]. Apart from the concentration effect, the chain length of amylose influences this phenomenon. Amylose with a degree of polymerization (DP) < 110 precipitates from aqueous solutions at all temperatures. Amylose of DP 250–660 precipitates or forms gel, depending on the concentration and temperature. For DP > 1100, amylose predominantly forms gels rather than precipitation [3].

Upon cooling, the starch suspension forms an elastic gel when the initial starch concentration is high. This involves interactions that occur mainly by hydrogen bonding among starch chains, a process known as retrogradation. In this process, amylose molecules start to be prone to double helix formation with the same type of molecules as well as with the long branches of amylopectin [7]. The amylopectin molecules also undergo a recrystallization process with the same type of molecules. As a result, gelatinized starch begins to re-associate in an ordered structure. In the case of a diluted starch suspension, the supernatant consists of mainly amylopectin. It may also contain highly branched non-amylopectin-type polysaccharides and small amounts of amylose. The precipitate contains mainly amylose and a part of branched amylopectin [3].

Although the gelation process as well as the starch gel itself has been studied extensively [8,9,10,11], not much attention has been given to studying the film formation process. Starch film preparation starting from a relatively dilute system (e.g., 1% starch in water which is a suspension) could lead to a different structural development during film formation compared to that of a relatively concentrated system (e.g., 5% starch in water which is a gel) since the physical status of these two systems are different at the initial stage. However, no previous in-depth studies on in situ starch film formation are available to compare these starch-solvent systems. Characterization of the starch films as obtained from these systems is important in order to understand and correlate the effect of initial concentration and possible changes in the molecular dynamics during film formation with the structural properties of the dried films which could help to tailor the films for already existing and new applications. There are several experimental techniques, for instance, atomic force microscopy (AFM), spectroscopic ellipsometry, X-ray photoelectron spectroscopy (XPS), X-ray diffractometry (XRD), and ultrasound reflection, that have been employed in order to follow the film formation of different polymer or inorganic materials [12,13,14,15]; however, none of them offer spatially resolved dynamical information as well as density profiling at the same time. In recent years, low-field [16,17,18,19,20,21] and high-field [22] NMR techniques have been employed successfully to study the film formation of different polymers and to characterize the prepared films [23]. In this contribution, a study of starch film formation is presented which follows the evolution from the initial polymer suspension (starting from a dilute system) or gel (starting from a concentrated system) to the final film using low-field NMR. The evolution of the molecular mobility of starch until the final stages of film drying was investigated with spatial resolution for the first time using NMR relaxometry, providing insight into the development of resolved polymer concentration.

## 2. Results and Discussion

### 2.1. Film Formation Study Using a Micro Camera

**Figure 1:**
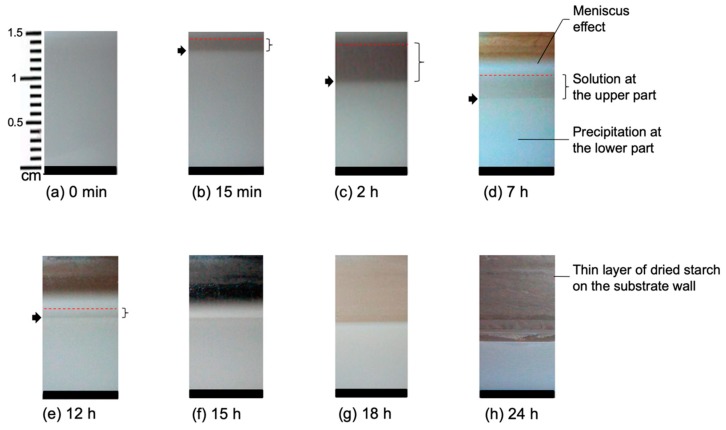
Sample *a* containing 1% starch in H_2_O at different stages of drying. The evolution time increases from (**a**) 15 min to (**h**) 24 h. The arrow shows the phase separation, whereas the bracket shows the upper solution layer. The different backgrounds in the upper part of the pictures are due to the different light at different times of the day.

A micro camera was used to follow Sample *a* containing 1% starch in H_2_O (*w/v*) and Sample *b* containing 5% starch in H_2_O (*w/v*), respectively, during real-time evaporation of the solvent. The camera was placed at a fixed distance from the Petri dish in the plane of the film, and pictures were taken at different drying stages. A set of images is shown in Figure 1 for Sample *a*. Figure 1a shows the fresh starch sample immediately after casting which appears homogeneous all over the sample height. After 15 min, two distinct layers become observable (see Figure 1b). The lower layer is a white, gel-like, and non-transparent substance. This layer contains mostly amylose which precipitates as explained in Section 1. On the other hand, the upper layer contains mainly amylopectin and relatively small amounts of amylose and highly branched polysaccharides. After 2 h of casting, as shown in Figure 1c, the separation of the layers is clearly visible, and the height of the lower layer is reduced. Figure 1d–f show a reduction of the height of the upper layer due to the evaporation of the solvent. There is a minor reduction of the height of the lower layer as well which indicates the continuation of packing of solute and removal of the solvent from the lower layer. In Figure 1g, it is seen that the upper layer of the sample is not detectable anymore after extended evaporation. After that, the lower layer continues to shrink with time as can be seen in Figure 1h. In this figure, a dried thin layer of starch can be observed on the wall of the Petri dish.

**Figure 2:**
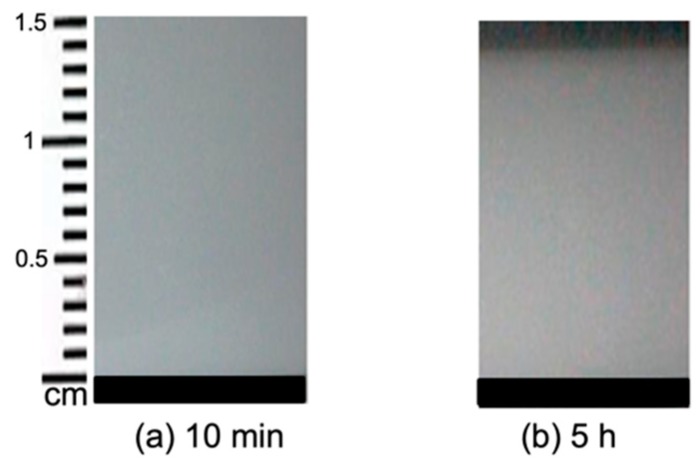
Sample *b* containing 5% starch in H_2_O at different stages of drying. The evolution time increases from (**a**) 10 min to (**b**) 5 h.

Figure 2 shows Sample *b* which was prepared using 5% (*w/v*) starch in H_2_O and cast on a Petri dish. The viscosity of this sample was high after gelatinization, and it formed a gel as can be seen in Figure 2. Due to the fact of retrogradation, amylose and amylopectin present in the system formed an ordered structure. As the solvent evaporated, the sample thickness decreased with time and the film was formed.

### 2.2. Real-Time Film Formation Study Using Single-Sided NMR Scanner

#### 2.2.1. Profiling of the Samples

When the height of the starch sample was approximately 1 mm following partial evaporation of H_2_O, it was placed onto the single-sided NMR scanning device. The starting time of the profile experiments was considered as t = 0 in the following discussion. For Sample *c*, this time corresponded to 78 h after the casting time, whereas 38 h had elapsed for Sample *d*. Seventy-eight hours after casting, water was already evaporated from the top layer of Sample *c* (corresponding to the phase rich in amylopectin) and became similar to Sample *a* in Figure 1h. On the other hand, Sample *d* was in the gel state after 38 h.

**Figure 3:**
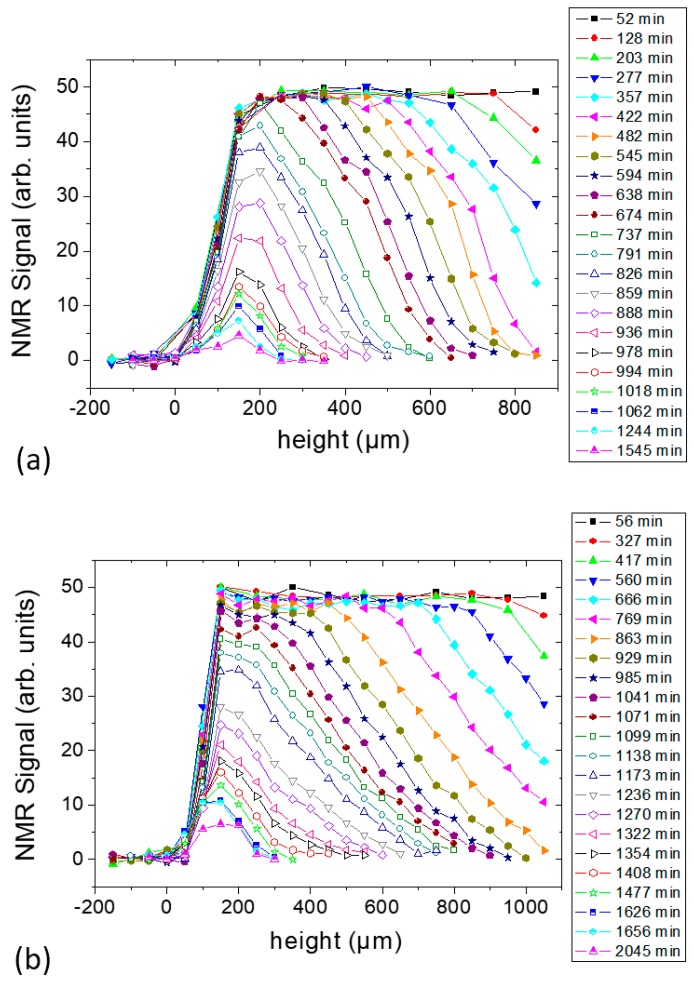
NMR profiles of the starch–water system at different drying times: (**a**) Sample *c* containing 1% starch in H_2_O (*w/v*) and (**b**) Sample *d* containing 5% starch in H_2_O (*w/v*). Each sample contained glycerol (20 g/100 g of starch) as a plasticizer.

For both samples, 23 consecutive experiments, i.e., complete profiles, were carried out as shown in Figure 3a,b for Samples *c* and *d*, respectively. The direction of scanning was from top (sample-air interface) to bottom (sample-substrate interface). The signal at negative height was obtained from the substrate only and was collected to define sample-substrate interface. The position of the sensor was moved in steps of 100 µm until 277 min and 417 min for Samples *c* and *d*, respectively, after which the step size was reduced to 50 µm. The figure shows that the initial height of the samples exceeds the scanning range of the sensor. The spatial resolution (i.e., the integration range) was fixed to 50 µm in both cases. For Sample *c*, with a pulse separation of 87.5 µs, 32 scans, 2048 echoes, and 8 s repetition time, a total time of ~256 s was required for the acquisition of data at one position, i.e., one individual slice. That means the time for scanning the full profile was ~52 min. This period was followed by a waiting time of 23 min. In Sample *d*, the repetition time was reduced to 7 s, and the number of scans was the same, so that each profile took 56 min to be completed; this period was followed by a waiting time of 34 min before the beginning of the subsequent slice.

With progressing solvent evaporation in both samples, less time was required to accumulate one profile as less repetition time was required at the later stage. The repetition time was always adjusted to be ≥5*T_1_* for the full recovery of the magnetization throughout the experiment. At the same time, the number of steps for each profile was reduced along with the reduction of the thickness. This permitted more acquisitions without increasing the time necessary to make a complete profile. Although Sample *d* contains a higher amount of starch and glycerol compared with Sample *c*, both experiments showed the same maximum intensity (within experimental error) in the initial profiles. A reduction in the signal intensity was observed after approximately 737 min and 929 min for Samples *c* and *d*, respectively, after being constant previously. This was due to the hardening of the sample with the corresponding decrease of the relaxation time, *T_2_*. It is important to mention that the first echo was forming at 87.5 µs [23]. For solid-like samples, a substantial portion of the signal decays inside this time as it was observed in the case of gelatin and PVOH films [16,17,18,19].

#### 2.2.2. Determination of Sample Shrinking Rate

**Figure 4:**
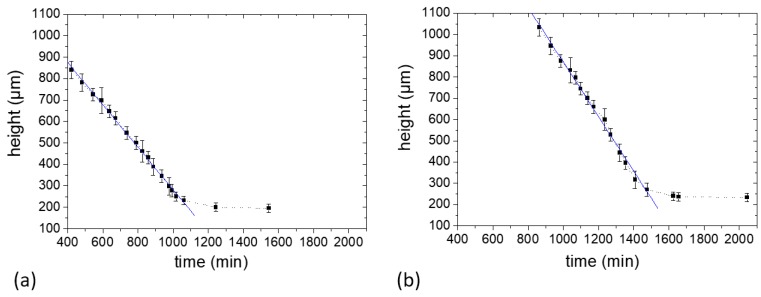
Film thickness estimated from the profile widths in Figure 3 as a function of drying time. Linear fits Table 1. (**a**) Sample *c* containing 1% starch in H_2_O (*w/v*) and (**b**) Sample *d* containing 5% starch in H_2_O (*w/v*). Each sample contained glycerol (20 g/100 g of starch) as a plasticizer.

The decrease in sample height as a function of evolving time is shown in Figure 4a,b for Sample *c* and Sample *d*, respectively. The sample height as calculated as the width of the profiles at 5% of the maximum intensity, where the signal can still be distinguished reliably from the noise level. With this assumption, the rate of evaporation (*Ė*) defined as the slope of the fitted lines was calculated for each sample.

At the beginning of drying, it was seen that the profile width decreases uniformly with time in both samples (Figure 4). These samples showed a single evaporation regime which indicates a continuous water evaporation during the experiment period. This tendency is comparable to previously studied gelatin biopolymer film formation system [23] and unlike the poly(vinyl alcohol) film formation system [19] where two distinct evaporation regimes were revealed. The vertical shrinkage rates calculated for Samples *c* and *d* were 1.6 × 10^−8^ m/s and 2.1 × 10^−8^ m/s, respectively. After 1070 min and 1480 min, respectively, there was no notable change in the profile widths of Samples *c* and *d* (within the experimental error) until the end of the NMR experiment as can be seen in Figure 4a,b. However, posterior shrinking was observed at time scales much longer, reaching final thickness of 130 µm and 170 µm for the Samples *c* and *d*, respectively. The films were stored in a desiccator (40 ± 5% RH, 21 ± 2 °C) for 3 days right after the NMR experiments to ensure complete drying of the films by measuring the thickness until a constant value was obtained. The evaporation period after the linear period was much slower, because it involved the release of water more strongly bounded in the system.

#### 2.2.3. Determination of *T_2_* at Different Heights

**Figure 5:**
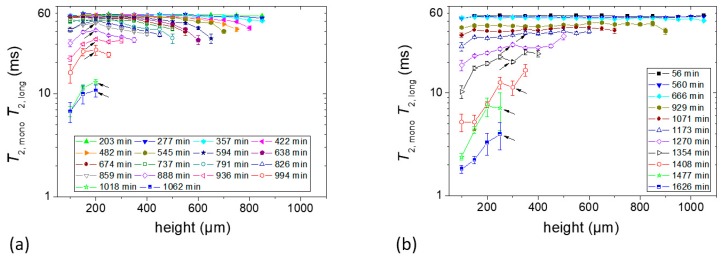
*T_2_* constants at different heights of the sample. (**a**) Sample *c* containing 1% starch in H_2_O (*w/v*) and (**b**) Sample *d* containing 5% starch in H_2_O (*w/v*). Each sample contained glycerol (20 g/100 g of starch) as a plasticizer. Layer positions correspond to coordinates introduced in Figure 3. At the later stage of drying, the echo decays were fitted with two exponential decay functions from the bottom of the sample to a certain height which is shown by the arrows. In the case of bi-exponential decay, the effective *T_2_* data shown in both figures correspond to the slowly decaying components.

Each point in the profile corresponds to the sum of the area of the second to fifth echo, whereas a much larger number of echoes was acquired for determining the transverse relaxation times *T_2_* layer by layer. For both samples, the Carr–Purcell–Meiboom–Gill (CPMG) echo decay [23,24] obtained from each profile point is mono-exponential at earlier evaporation times. Accordingly, a mono-exponential function was fitted to the experimental data to measure *T_2_*. At a later stage of drying, the decay curves obtained from the bottom to a certain height of the sample required a fit with two exponential decays. Figure 5a,b show the *T_2_* values obtained either from the single component at an earlier stage, or a combination of single and slowly decaying components (long component) at the later stage during the total drying time with respect to different positions in the Sample *c* and Sample *d*, respectively. In general, both samples show nearly the same *T_2_* values (~60 ms) at the beginning of the experiment as can be seen in Figure 5. Note that the measured decay constants represent the contribution of true relaxation, *T_2_*, as well as decay due to the self-diffusion in between the rf pulses, according to the following equation [25]:

(1) $$\frac{1}{T_{2,\;eff}}=\frac{1}{T_{2}}+\frac{\gamma^{2}G_{z}^{2}\tau_{echo}^{2}}{12}D$$

where γ is the magnetogyric ratio; *G_z_* is the magnetic field gradient along *z*; *τ_echo_* is the separation between spin echoes, hence between rf pulses; and *D* is the self-diffusion coefficient.

With the assumption of the bulk water self-diffusion coefficient (*D* = 2.3 × 10^−9^ m^2^/s at 25 °C, the actual value in the gel is probably slightly smaller) and an echo time of 87.5 µs, the contribution of the second term is estimated as 13.9 s^−1^, compared to the measured value of *T*_2,eff_^−1^ of about 16.6 s^−1^. The actual *T_2_* is thus expected to be around 400 ms with a large uncertainty. Consequently, for the early times during evaporation, the measured *T_2_* is actually the apparent or “effective” *T*_2,eff_ which begins to decrease only after the diffusion contribution decreases due to the lower diffusion coefficient and when the real *T_2_* becomes smaller than the diffusion contribution.

The *T_2_* values decreased with the time of film formation in both starch samples until the end of the measurement period. This is attributed to the film formation process and solvent evaporation. Two types of dependencies of *T_2_* values on the position within the Sample *c* were observed during the evaporation process as can be seen in Figure 5a. Until approximately 890 min of drying, the *T_2_* values gradually decreased from the bottom towards the sample–air interface which followed a definite tendency (see below). The first *T_2_* profile showed that there was a slight decrease in *T_2_* values (~60 ms to ~55 ms) with the increase of height. This difference became more pronounced at later stages of evaporation. Another observation in this sample was that the change in *T_2_* values was initially less pronounced with the increase of sample height and drying time at the bottom part of the sample. The gradient of *T_2_* over evolution time was obviously much larger in the top 200 µm at any time up to approximately 740 min of drying (Figure 5a). From 888 min onwards, there was a gradient in the dependence of *T_2_* on the height of Sample *c* which shows that the bottom part of the sample progressively developed the lowest *T_2_* values.

The *T_2_* value of Sample *d* for different heights of the first *T_2_* profile was ~56 ms, as can be seen in Figure 5b, and remained almost unchanged until after 666 min, when it began to vary from layer to layer in the sample, as shown in Sample *c*, at the later stage of drying but with an opposite tendency: relaxation becomes shortest at the bottom from the very beginning.

From 1354 min onwards, *T_2_* values decreased sharply in the bottom part of the sample. For example, they underwent a two- to three-fold decrease in a short interval until 1408 min.

**Figure 6:**
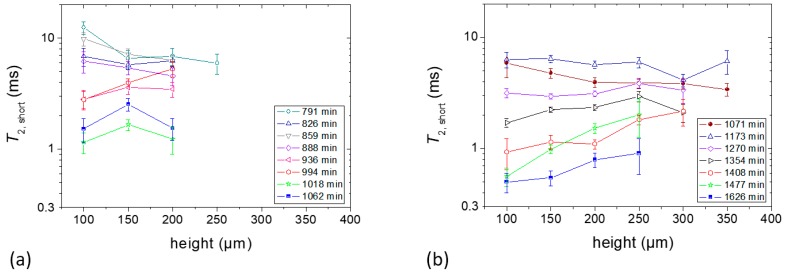
*T_2_* values of the fast decaying components at different heights of the sample. (**a**) Sample *c* containing 1% starch in H_2_O (*w/v*) and (**b**) Sample *d* containing 5% starch in H_2_O (*w/v*). Each sample contained glycerol (20 g/100 g of starch) as a plasticizer. Layer positions correspond to coordinates introduced in Figure 3.

Both samples showed a second component during the later stage of film formation (after 791 min and 1071 min of drying time for Samples *c* and *d*, respectively) from the bottom to a certain height of the sample. Figure 6a,b show the short *T_2_* values (designated as *T_2,short_*) obtained from the fast decaying components with respect to different positions in Samples *c* and *d*, respectively. In Sample *c*, the highest *T_2,short_* value of the fast decaying component was ~13 ms after 791 min of drying time which was reduced to ~1 ms after 1062 min. In Sample *d*, on the other hand, the *T_2,short_* value decreased from ~6 ms to ~0.5 ms in between 1071 min and 1626 min of the experiment time. The *T_2,short_* values in Sample *d* showed the same type of depth dependence as was found for the slow decaying components in Figure 5b, whereas the variation for the slow decaying components of Sample *c* remained comparable to the shown error in Figure 6a.

As described in Section 2.2.1, Sample *c* formed a suspension, whereas Sample *d* was a gel immediately following casting. To correlate this observation with the *T_2_* experiments, one can see that the molecular mobility in the vicinity of the air–sample interface in Sample *c* decreased during evaporation at an earlier stage. This effect was absent in Sample *d*. At a later stage, it was observed in both samples that the mobility increased with the sample height, especially in the sample containing 5% starch (i.e., Sample *d*).

For the repetition samples using H_2_O as a solvent, similar profiling of the film was carried out using the single-sided NMR scanner to follow the drying of the starch suspension. Detailed analysis of the data was done in similar fashion, and an identical trend of starch film formation behavior was observed from the *T_2_* values.

#### 2.2.4. Determination of *T_2_* of Starch Samples at an Early Stage

**Figure 7:**
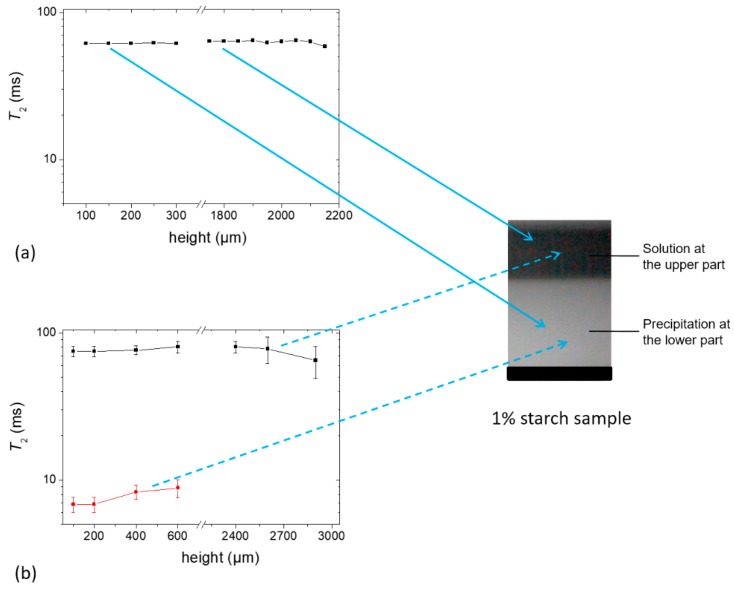
*T_2_* constants at different heights of (**a**) Sample *e* containing 1% starch in H_2_O (*w/v*) (echo decay was collected using 32 scans) and (**b**) Sample *f* containing 1% starch in D_2_O (*w/v*) (echo decay was collected using 2048 scans). Glycerol (20 g/100 g of starch) was used as a plasticizer.

Aqueous suspension of 1% starch sample was prepared using an H_2_O (Sample *e*) or D_2_O (Sample *f*) as a solvent. Each sample was placed on top of the single-sided NMR scanner when a precipitation was clearly visible within 1 h of waiting time. For this study, the scanner was set to allow an accessible vertical range of 4.1 mm to obtain the *T_2_* of both of the upper and lower layers using the CPMG pulse sequence [23]. Figure 7a,b show the measured *T_2_* values for Samples *e* and *f*, respectively. Thirty-two scans were sufficient to obtain an echo decay with an acceptable signal-to-noise ratio in the case of Sample *e*, whereas 2048 scans were required for Sample *f*. Note that the echo decay signal intensity of Sample *f* was approximately 35 times lower than that of Sample *e*. It was seen that the *T_2_* values were nearly homogeneous at different heights of the sample. The *T_2_* value of the upper part of Sample *e* was ~60 ms which is nearly the same as the *T_2_* values of Sample *c* and *d* at the beginning of the profile experiment as can be seen in Figure 5. The higher value of *T*_2,*eff*_ in Sample *f* (~75 ms compared to ~60 ms in Sample *e*) can be understood from Equation (1) since the diffusion co-efficient of D_2_O (D_D2O_ =1.87 × 10^−9^ m^2^/s at 25 °C) was approximately 20% lower than that of H_2_O. With time, mobility decreased in the sample and this effective *T_2_* value approached the real value.

From Figure 7b, it can be seen that the fresh Sample *f* shows the *T_2_* relaxation of two components. The lower precipitation part of this sample showed a *T_2,short_* of ~7 ms, which increased somewhat with sample height, and a *T_2,long_* of ~75 ms. The short component must therefore have its origin in less mobile protons of starch which were completely masked by the water phase in Sample *e*. On the other hand, the upper solution part showed only one component with a *T_2_* of ~75 ms which consisted of the contribution from water and mobile fractions in the sample. Note that this sample probably contained HDO molecules due to the exchangeable protons from the starch polymer [26] and the glycerol plasticizer which may have contributed to the higher *T_2_* value. Taking into account that the concentration of glycerol in the total sample was approximately 0.2% at the beginning and 3 protons among 8 protons of glycerol are exchangeable, the percentage of protons in the glycerol was significantly lower than the total residual protons in the sample, and thus the contribution of glycerol to the signal was negligible. However, it is worth mentioning that this minute amount of glycerol could affect the dynamics of the starch/glycerol system at a later stage in the dried film (see below).

For the repetition of samples using D_2_O as a solvent, two *T_2_* relaxation components with similar values were confirmed.

### 2.3. Dried Film Characterizations

The final films obtained from Samples *c* and *d* were stored keeping the same environmental conditions of the drying process for two weeks and were then measured using NMR and XRD techniques.

#### 2.3.1. Single-Sided NMR Study

**Figure 8:**
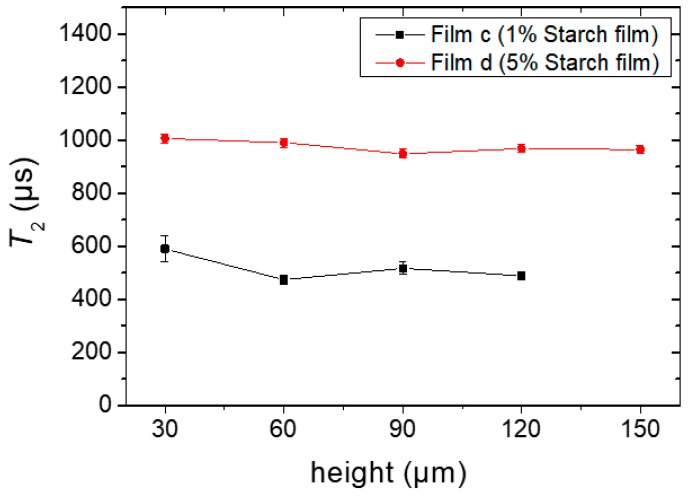
*T_2_* constants at different heights of the completely dried films *c* and *d*, prepared from Sample *c* containing 1% starch in H_2_O (*w/v*) and Sample *d* containing 5% starch in H_2_O (*w/v*), respectively. Glycerol (20 g/100 g of starch) was used as a plasticizer during film preparation.

The *T_2_* experiments were performed for the films obtained from Sample *c* (named as film *c*) and Sample *d* (named as film *d*), respectively, and the results are shown in Figure 8. As the resolution of 30 μm was set for this study to obtain the maximum number of experimental data points, the corresponding echo time was set to 114.5 µs (see experimental Section 4.2.4). *T_2_* was uniform within the experimental error when the film was in its final stage, despite dynamic heterogeneities during the drying process. Short *T_2_* components (faster decays) were expected in these films. As a comparison, in an earlier study on gelatin biopolymer film formation [16], it was shown that a fast decaying component (characteristic of solid like signals) could be distinguished in the dried film sample which decayed below 100 µs. This signal could not be detected in the single-sided NMR device. Therefore, the *T_2_* values shown in Figure 8 represented only the longer component of the transverse relaxation times of a solid film, and the presence of shorter *T*_2_ components cannot be discarded.

The values of *T_2_* were ~550 µs and 1 ms for film *c* and *d*, respectively. Even when one expects different relaxation characteristics for different biopolymers in the film state, it is instructive to compare starch with the case of gelatin. The *T*_2_ values for starch films were significantly longer than those found in gelatin films (200–300 µs) [17], mainly due to the presence of the non-volatile glycerol acting as a plasticizer which contributes to the retention of a certain degree of molecular mobility in the whole structure. The initial glycerol content in the total system (0.2% in Sample *c* compared with 1% in Sample *d*) is reflected in the difference of the *T_2_* values, where, for the case of Sample *d*, a higher residual mobility and longer *T*_2_ values were observed.

The residual water contents in films *c* and *d* were determined after the samples were oven-dried at 105 °C until a constant weight was approached. The moisture contents (dry weight basis) in both films were in the order of 11% (Sample *c*) and 14% (Sample *d*), respectively. This difference correlates with the longer value of *T_2_* for Sample *d* with more residual humidity, probably due to the higher glycerol content.

#### 2.3.2. X-Ray Diffraction (XRD) Study

**Figure 9:**
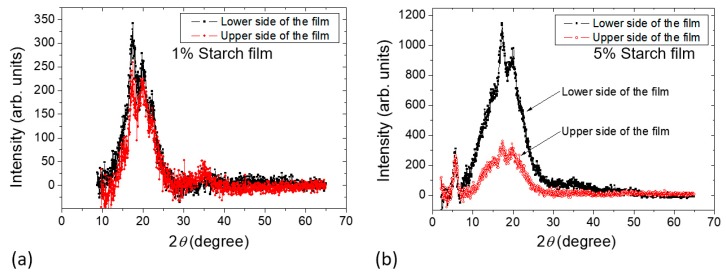
X-ray patterns of films prepared from (**a**) Sample *c* containing 1% starch in H_2_O (*w/v*) and (**b**) Sample *d* containing 5% starch in H_2_O (*w/v*).

The X-ray diffractograms of the dried films obtained from Samples *c* and *d* are shown in Figure 9. The amorphous contribution was subtracted [27]. The measurements were done at a fixed incoming low incident angle. In this way, the X-ray beam spot spreads on a relatively big surface on the film, reducing the X-ray flux on the sample [18,28]. In this configuration, X-rays will also be less likely to penetrate through the sample, and the signal will carry, at most, the structural information of the side of the sample facing the incidental X-ray beam. Assignment of the specific diffraction peaks compared with the XRD spectra presented in Le Bail et al. [29] suggests that the starch films obtained from the film formation study contain B-type crystallinity. This observation is in agreement with the studies described in the literature, for example, in Reference [30], where B-type crystallinity was observed in starch films prepared from corn starch. Note that B-type crystallinity was found in some tuber and cereal starches rich in amylose. The B-type pattern has relatively less densely packed double helices than the A-type pattern. Figure 9a shows that both sides of the XRD pattern for Sample *c* are nearly the same, whereas the film obtained from Sample *d* shows (Figure 9b) that the lower side of the film has a higher intensity in the peak compared with that of the upper side of the film. In Figure 9b, the significant fact is that the peak at 2θ ~ 5.6° shows the same intensity for both sides of the film obtained from Sample *d*, although there is a significant difference in intensity in the peaks at 2θ ~ 17° and 20° when both sides are compared. This can only be explained by assuming that the sample contains a higher fraction of amorphous domains in the upper side. This agrees with the NMR relaxation experiments showing that during the film formation at a later stage in Sample *d* (Section 2.2.3), a decrease in mobility of the sample was observed at the lower part revealed by the increase of *T_2_* relaxation time with sample height. The XRD data further complement the *T_2_* effect as observed during the film formation study.

## 3. Proposed Mechanism of Starch Film Formation

In this study, two different tendencies in the molecular mobility were observed during the starch film formation processes depending on the initial concentration. The mechanism that accounts for these observations takes place necessarily during the film formation. It is known that when the semi-crystalline structure of the starch granule is treated in hot water, the linear amylose can diffuse through the granule more readily than the highly branched amylopectin. When the temperature of the system is reduced, the more diluted starch solution (1% in the present case) forms a precipitate which contains mainly amylose. In this diluted system, amylose units form very compact double helices that further associate to form the precipitate [7,31]. Amylopectin and highly branched polysaccharides take part in the precipitation as well to a lesser extent since the amylose forms complex structure with these molecules [3]. The highly branched molecules are more concentrated in the supernatant part because of their affinity to water. Exchange due to the fact of diffusion at the bottom part of this region between amylose and amylopectin molecules is taking place as well. Before a concentrated layer rich in amylopectin is formed after the water from the supernatant has completely evaporated, a gradient in the dynamics towards the sample–air interface is established along the sample due to the evaporation of water. The profile of the relaxation times in Figure 5a expresses the signature of a gradual loss of mobility in the upper layers. This can be explained due to the fact that in diluted solutions, the evaporation of the solvent induces a concentration profile [32], where the maximum concentration of the solute is located at the evaporating surface. The correlation between concentration of solute and viscosity provides the explanation that molecular mobility is more restricted, and therefore the relaxation times are shorter close to the outer layers of the sample. Similar phenomena were reported in the film formation of gelatin in water solution at low initial concentrations (<2%) [17]. The starch sample prepared from 5% starch in water is a gel from the beginning of film formation which does not show this tendency because overall molecular migration of starch is prohibited.

**Figure 10:**
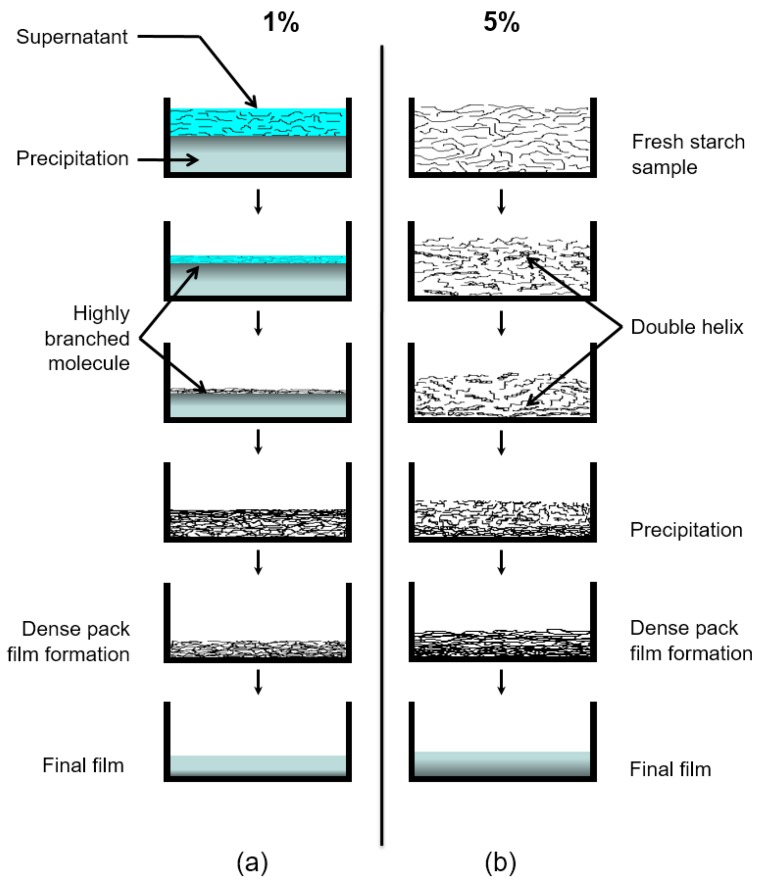
Illustration of a proposed mechanism for the starch film formation system at different stages of drying times. (**a**) Sample *c* containing 1% starch in H_2_O (*w/v*) and (**b**) Sample *d* containing 5% starch in H_2_O (*w/v*). The evolution time increases from top to bottom.

At a later stage, a decrease in *T_2_* at the bottom of the sample can be observed in both systems, especially in the 5% starch-containing sample. Note that starch gel consists of double helical crystallites [3]. In the present case, the crosslinked gel system is not stable due to the continuous evaporation of water. Consequently, it is most likely that the double helix structure precipitates when the water concentration is reduced in the system, assuming that the water takes part in the structure making hydrogen bonds. This process reduces the mobility in the bottom part of the sample which is possible to follow from the relaxation study. In this way, the interplay of water evaporation and polymer migration results, for the conditions studied in this work, in a dynamic heterogeneity of the polymer molecules across the film thickness which develops gradually after the average water concentration drops below a critical value. Figure 10 illustrates the possible mechanism as described above. The XRD data show that the dense triple helix structure that is preferably generated at the bottom of the sample during the evaporation makes a significant difference in the structure of the final film.

## 4. Materials and Methods

### 4.1. Materials

Unmodified extra pure corn starch containing ~13% moisture (dry weight basis) was obtained from Carl Roth GmbH + Co. Mono-distilled water was obtained from the GFL-2002 water distillation unit (Gesellschaft für Labortechnik mbH, Germany) and used to dissolve the polymer samples. Deuterated water (100 atom-% D) was used as a solvent in order to selectively follow the dynamics of the polymer molecules during the NMR experiments and was purchased from Chemotrade, Chemiehandelsgesellschaft mbH, Leipzig, Germany. Films prepared from pure starch are often very fragile. Hence, low molecular weight plasticizers, such as polyols, are used to decrease interactions among the polymer chains [33,34,35]. In this study, anhydrous glycerol (Merk-Schuchardt, Germany) was used as a plasticizer. It is generally used to enhance the flexibility and elasticity of the starch film as well as to prevent pore and crack formation [35].

### 4.2. Methods

#### 4.2.1. Sample Preparation

Starch contains ~13% moisture which can contribute to the total NMR signal. The moisture was removed by oven-drying at 105 °C. It was then kept in a desiccator in moisture-free atmosphere at ambient temperature for storage. Aqueous dispersions of 1% and 5% (*w/v*) corn starch were prepared in presence of glycerol (20 g/100 g of starch) plasticizer. The protocol for sample preparation was the following: A precalculated amount of starch and solvent (H_2_O or D_2_O) mixture was heated and stirred at 70 °C for 40 min in a covered beaker to obtain a homogeneous mixture. The starch dispersion was then heated to nearly 95 °C and kept at this temperature for about 20 min to allow gelatinization. It was observed that the 1% starch sample formed a precipitate when it was allowed to cool without agitation. On the other hand, the 5% starch sample formed a white gel [6]. Consequently, both samples were agitated to maintain a homogeneous mixture until the sample temperature was reduced to approximately 40 °C and cast afterwards. Polystyrene Petri dishes of 55 mm diameter, which are non-adhesive to starch, were used to cast the samples. This allows one to peel out the final dry film for further studies. All cast samples were kept at the same room humidity and temperature (40% ± 5% RH, 21 ± 2 °C) until each film was completely dried and characterized using different techniques.

#### 4.2.2. Samples

Table 1 summarizes the samples used in this study. A micro camera was used to follow the reduction of the sample height due to the evaporation in Samples *a* and *b*. Samples *c* and *d* were used to study the film formation in the single-sided NMR scanner. A larger portion, as optimized from a number of film preparation trials, of the lower-concentration Sample *c* was cast on the Petri dish compared to that of the higher-concentration Sample *d* to obtain a final film thickness of at least 100 µm. Consequently, the evaporation time of Sample *c* was longer than that of the other samples. For Samples *e* and *f*, the *T_2_* values of the samples were obtained using an accessible vertical range of 4.1 mm of the single-sided NMR scanner which will be discussed below. Experiments involving Samples *c*–*f* were repeated for data reproducibility using a separate set of samples; however, experiments for Samples *a* and *b* were conducted only once.

#### 4.2.3. Film Formation Study Using a Micro Camera

A digital microscopic camera (Somikon, Pearl Agency Allgemeine Vermittlungsgesellschaft mbH, Germany) was employed to capture real-time still pictures at different stages of starch film formation to visually compare different samples.

#### 4.2.4. Film Formation Study Using the Single-Sided NMR Scanner

The polymer film formation was followed using a single-sided NMR scanner [23,36,37], the so-called profile NMR MOUSE (MObile Universal Surface Explorer, NMR MOUSE PM10, ACT GmbH, Germany). The device was operated at a proton (^1^H) Larmor frequency of 11.7 MHz with a well-defined constant field gradient of 11.5 T/m. The sensor has a sensitive area of about 10 mm by 10 mm. The distance between the magnet and the RF coil determines the accessible sample height [36]. Most of the measurements in this work (except for that of Samples *e* and *f*) were carried out using the minimum distance between the sensitive volume and the coil, allowing an accessible vertical range of 2.1 mm. The Petri dish containing the precalculated amount of aqueous polymer sample was placed on a fixed platform on top of the movable sensor to follow the film formation process. When the magnet is moved in the vertical direction using a precision lift controlled by a step motor, the RF coil as well as the sensitive volume move accordingly through the sample and a profile [37] can be obtained. Because of the inhomogeneity of the magnetic field, the characteristic transverse relaxation time T_2_^*^ of the FID is very short (in the order of few µs) and cannot be detected in this scanner. Instead, with the application of CPMG pulse sequence, one can overcome this problem because the FID is refocused at a time that is longer than the dead time. Hence, the CPMG pulse sequence was applied to obtain the vertical profiles and to accumulate *T_2_*-weighted echo trains by moving the scanner relative to the sample as well as to measure the effective time of echo decays. Accordingly, *T_2_* relaxation time data obtained from the single-sided scanner will be considered as the effective *T_2_* time. The 90° RF pulse length was set to 3.5 µs after calibration and was the same for all samples.

In the scanner, the resolution was set by controlling the acquisition time *T*. This approach requires to set the RF pulse to the shortest duration to maximize the excited region and then to set the acquisition time to achieve the desired resolution inside the excited slice (by acquiring signal during a time *T* the frequency resolution in the spectrum is Δν=1/T). The thickness of the selected slice was Δz=2π Δν/γG, where *γ* and *G* are the magnetogyric ratio and field gradient, respectively [37]. Thus, for the slice thicknesses of 30 and 50 μm, the 180° pulse separations were set to 114.5 and 87.5 µs, respectively, taking the deadtime of 17 µs into account.

#### 4.2.5. Dried Film Characterizations

Prior to the measurements, the samples were stored in the same environmental conditions as employed for the NMR experiments during film formation. The *T_2_* values of the fully dried films were measured as a function of height using the CPMG pulse sequence using the single-sided NMR scanner. The XRD patterns of both sides of the same starch film were acquired with a Philips X’Pert PRO diffractometer to examine the structural details. The diffractometer was equipped with a wide-range PW 3050/6X goniometer which was capable of measuring 0.001°/step. The experiments were carried out at room temperature using Cu-Kα radiation (λ = 0.154 nm) generated at a voltage of 35 kV and 30 mA current. The fixed-angle incident beam method [18] was used, where Ω = 0.5° was fixed throughout the experiment. The samples were scanned between 2θ = 1.5° and 65° with a step size of 0.050° and a scanning speed of 2°/min.

## 5. Conclusions

A number of studies were conducted to follow the evaporation of the solvent from two different starch samples containing 1% and 5% of starch in water (*w/v*) during the process of film formation. These studies enable one to understand the starch–H_2_O system (in the presence of plasticizer) at a molecular level. Using a micro camera, evaporation of the solvent from these starch systems was observed optically, revealing that the evaporation tendency of these two systems is different. While the sample containing 1% starch formed a precipitate at the beginning, the sample containing 5% starch formed a gel. With evaporation, the thickness of the upper solution part of 1% starch sample was reduced due to the fact of solvent evaporation, and the lower precipitation layer became densely packed.

The 1% starch-containing sample showed a gradual decrease in the mobility of the polymer molecules near the air–sample interface at the beginning of the experiment which was totally absent in the sample containing 5% starch in water. When both starch samples were approaching the final drying stage, the *T_2_* values near the substrate decreased more strongly compared with the region in the vicinity of the air–sample interface. Subsequent analysis of the completely dried film by X-ray diffractometry revealed that the amorphous fraction in the region at the upper surface was indeed considerably higher than that at the lower surface. These observations were similar to earlier findings obtained for gelatin solutions [16,17], where network formation and precipitation resulted in an increase of the crystalline domains at the bottom surface. However, the distribution of amorphous/crystal domains in the case of other films, for instance, for the (water soluble) PVOH films (see Reference [19]), is inverted. One obvious reason for this is the different chemical composition and architecture of the polymer/biopolymer, but perhaps the more important fact is the relative interplay of overall polymeric reorientation timescale on the one hand and the corresponding water/polymer interaction on the other hand which varies in dependence of time as water is removed from the system.

The different dynamical and structural features found in starch films possess the potential for optimized and designed manufacturing processes by carefully controlling initial concentration, additives, and evaporation conditions.

## Figures and Tables

**Table 1:** Different starch samples prepared using H_2_O or D_2_O as a solvent. Glycerol was added as a plasticizer (20 g/100 g starch) to prepare each sample.

Sample	Initial Concentration (%) of Starch in the Solvent (*w/v*)	Solvent	Initial Physical Status of the Sample	Study	Thickness of the Final ^3^ Film (µm)
*a*	1	H_2_O	suspension	Using micro camera	-
*b*	5	H_2_O	gel	Using micro camera	-
*c*	1	H_2_O	suspension	Profiling ^1^ at 11.7 MHz (^1^H) and XRD	130 ± 15
*d*	5	H_2_O	gel	Profiling ^2^ at 11.7 MHz (^1^H) and XRD	170 ± 10
*e*	1	H_2_O	suspension	*T_2_* study at 11.7 MHz (^1^H)	-
*f*	1	D_2_O	suspension	*T_2_* study at 11.7 MHz (^1^H)	-

^1,2^ At the beginning of the NMR profiling experiments, these samples had evolved to their gel state. ^3^ The films were kept in a desiccator cabinet for 3 days after the NMR experiments to ensure complete drying and constant final thickness.
